# Reduction of Trimethylamine Off-Odor by Lactic Acid Bacteria Isolated from Korean Traditional Fermented Food and Their In Situ Application

**DOI:** 10.4014/jmb.2005.05007

**Published:** 2020-06-24

**Authors:** Seul-Ki Park, Du-Min Jo, Daeung Yu, Fazlurrahman Khan, Yang Bong Lee, Young-Mog Kim

**Affiliations:** 1 Institute of Food Science, Pukyong National University, Busan 48513, Republic of Korea; 2 Department of Food Science and Technology, Pukyong National University, Busan 48513, Republic of Korea; 3 Department of Food and Nutrition, Changwon National University, Changwon 51140, Republic of Korea

**Keywords:** TMA reduction, lactic acid bacteria, deodorization, phylogenetic tree, seafood, in situ application

## Abstract

Trimethylamine (TMA) is a well-known off-odor compound in fish and fishery products and is a metabolic product of trimethylamine *N*-oxide (TMAO) generated by the enzymatic action of microorganisms. The off-odor is a factor that can debase the value of fish and fishery products. The present study aimed to remove TMA using lactic acid bacteria (LAB). A total of fifteen isolates exhibiting the TMA reduction efficacy were isolated from Korean traditional fermented foods. Among these isolates, five LAB isolates (*Lactobacillus plantarum* SKD 1 and 4; *Lactobacillus paraplantarum* SKD 15; *Pediococcus stilesii* SKD 11; *P. pentosaceus* SKD 14) were selected based on their high TMA reduction efficacy. In situ reduction of TMA efficacy by the LAB cell-free supernatant was evaluated using a spoiled fish sample. The results showed effective TMA reduction by our selected strains: SKD1 (45%), SKD4 (62%), SKD11 (60%), SKD14 (59%), and SKD15 (52%), respectively. This is the first study on TMA reduction by the metabolic activity of LAB and in situ reduction of TMA using cell-free supernatant of LAB. The present finding suggests an economically useful and ecofriendly approach to the reduction of TMA.

## Introduction

Trimethylamine (TMA) is an organic and volatile fatty amine known to cause acute health problems such as pulmonary stimulation, eye irritation, and liver failure [1]. The source of TMA is a nitrogenous organic compound named trimethylamine *N*-oxide (TMAO). TMAO is an oxidized form of TMA isolated from seawater and known to present in large quantities in fish muscle and tissue [2-4]. Most marine fish and shellfish have mechanisms for digesting TMAO, usually by the osmoregulation process. Similarly, in frozen saltwater fish, TMAO is reduced to dimethylamine (DMA) and formaldehyde products by the action of endogenous enzymes, whereas TMAO content in fresh or iced fish is reduced to trimethylamine (TMA) by the metabolic activity of bacteria and bacterial enzymes [2, 5, 6]. Previous studies reported that several bacterial species such as *Pseudomonas* sp., *Vibrio* sp., and *Bacillus* sp. have properties that metabolically convert TMAO into TMA [7]. Being volatile, TMA constitutes one of the major components of fish off-odor. Hence, in the current scenario, the detection of TMA off-odor released from fish or shellfish is used as an indicator of their freshness. Previous studies reported several methods for the reduction of TMA such as microbubble treatment using supercritical CO_2_ [8], application of an active charcoal [9-11], and physio-chemical extraction process [12, 13]. Similarly, another approach for the reduction of TMA is the re-oxidation of TMAO by the action of TMA monooxygenase (TMO) present in mammals and microorganisms [14]. The TMO enzyme produced by organisms such as *Aminobacter aminovorans*, *Roseovarius* sp., *Homo sapiens*, and *Methylocella silvestris* is registered in the BRENDA enzyme portal (Braunschweig Enzyme Database, http://www.brenda-enzymes.org). Lidbury *et al*. [15] reported that *Roseobacter pomeroyi* utilized TMA and TMAO for the synthesis of energy in the form of ATP, which supports cell growth and survival. In the diverse range of food products, there are numerous beneficial bacterial species, such as lactic acid bacteria (LAB), as well as non-beneficial bacterial species that are known to exist. Hence, the isolation of beneficial bacterial species with TMA reduction properties would be considered economically useful and ecofriendly. Thus, based on the available information, the present study aimed to isolate and identify LAB with metabolic activity for the reduction of TMA. Furthermore, in the present study, these potential LAB isolates were also successfully applied for in situ reduction of the TMA content in spoiled fish samples.

## Materials and Methods 

### Screening of LAB from Korean Traditional Fermented Food

Various Korean traditional fermented foods were screened for LAB. Samples were obtained from local markets in Busan, Republic of Korea. The samples included one type of meju (fermented soybean), 2 types of makgeolli (rice wine), 1 type of kimchi, and fermented kelp. For screening of LAB, 25 g of samples were added in a 250 ml Erlenmeyer flask containing 125 ml of 0.1% (w/v) peptone water. The mixture was homogenized and serially diluted up to 10^-5^ dilution. After that, serially diluted samples were spread plated on bromo cresol purple agar (BCP, plate count agar with 0.006% bromo cresol purple) plates and incubated at 30°C. The LAB produced several organic acids that reduced the pH and turned the medium yellow. We then used these yellow colonies for further testing [16].

### Identification of Isolated LAB

The isolated colonies were identified by using the 16S rRNA sequencing and biochemical method. Genomic DNA of LAB isolates was prepared using the AccuPrep Genomic DNA Extraction Kit (Bioneer, Republic of Korea). Amplification of 16S rRNA genes from the LAB isolates was done by PCR using universal primers 27F (5’-AGAGTTTGATCCTGGCTCAG-3’) and 1492R (5’-TACGGYTACCT TGTTACGTACTT-3’) [17]. The obtained 16S rRNA gene sequences of the LAB isolates were deposited in the GenBank nucleotide sequence database. The sequences were determined with the Basic Local Alignment Search Tool (http://ncbi.nlm.nih.gov/BLAST), which was used to find homologous sequences. Furthermore, a phylogenetic tree was constructed to determine the closest bacterial species using the neighbor-joining method of MEGA 7 (ver. 7.0.26). The carbohydrate fermentation pattern and biochemical identification of the LAB isolates were studied using the API 50 CH and API 50 CHL system (Bio-Merieux, France) following the manufacturer’s protocol.

### TMA Reduction Efficacy of LAB Isolates 

To prepare the LAB cell-free culture, the LAB was cultivated in DeMan-Rogosa-Sharpe (MRS) medium at 37°C up to about 9 log CFU/ml. The LAB cell-free culture was obtained by aseptic filtration of the supernatant using a 0.2 μm membrane (Toyo Co., Japan) after low centrifugation (10,000 g, 20 min). TMA reduction efficacy of LAB was carried out by mixing cultured LAB cell-free medium and TMA (0.3% of final concentration, v/v). We measured the TMA reduction efficacy of this LAB/TMA mixture after 5 and 24 h reactions at 25°C. TMA reagent was purchased from Junsei Co. (Japan). The LAB cell-free medium and TMA mixture was moved into 22 ml, clear glass vials with polytetrafluoroethylene (PTFE)-silicone septa and open-top phenolic caps and vortexed before TMA extraction and gas chromatography (GC) analysis.

### TMA Extraction and Quantitative Analysis

TMA contents were extracted and quantitatively determined using the GC Solid Phase Microextraction (SPME) method as described by Park *et al*. [18] with a slight modification. Briefly, TMA was extracted in a 22 ml vial at 70°C and 200 rpm for 20 min. SPME fiber assemblies, fiber holders (needle size of 24 ga) for manual use, 65 µm PDMS/DVS were purchased from Supelco Co. (Bellefonte, USA). Then, samples were analyzed using GC with flame-ionization detection (FID) (6890N, Agilent Technologies, USA) with SPME. The separation was carried out on the HP-5 column (30 m × 0.320 mm; id, 0.25 µm) at detector and injector temperature of 250°C in split-less mode. Flow rates of carrier gases were 30, 25, and 300 ml/min for nitrogen, hydrogen, and air, respectively. Oven temperature was held at 5°C (initial holding condition) for 5 min and increased up to 150°C at a rate of 10°C/min with a holding at 15°C for 15 min. Finally, the oven temperature was increased and held at 250°C for 5 min for the cleaning process.

### In-Situ Reduction of TMA by Cell-Free Supernatant of LAB Culture

To validate the TMA reduction ability of the LAB isolates, we performed in situ reduction testing by augmenting cell-free supernatant of LAB cell culture onto the spoiled fish samples. The fish samples (Ribbon fish; *Trichiurus lepturus*) were purchased from a traditional market in Busan, Korea. The cell-free supernatant of LAB was prepared by growing in MRS medium at 37°C up to about 9 log CFU/ml. The obtained cell culture was centrifuged (10,000 g, 20 min) and filtered using a 0.2 μm membrane (Toyo Co.). To prepare the spoiled fish samples, whole fishes were homogenized, and the samples were then sufficiently spoiled at 25°C for 48 h. Then, 4 ml of each LAB cell-free culture was vortexed with 1 g of spoiled fish samples, and the TMA was immediately extracted and analyzed as described above.

### Statistical Analysis

All experiments were carried out in triplicates. Data were expressed as mean ± standard deviation. Obtained data were statistically analyzed by analysis of variance (ANOVA) and Duncan’s multiple range test. Statistical significance of differences between mean values of data was interpreted at a significance level of *p* < 0.05.

## Results and Discussion

### Identification of LAB Isolates from Korean Fermented Food

Fifteen LAB isolates were isolated and identified from four types of Korean traditional fermented foods. All fifteen LAB isolates were identified by PCR amplification and sequencing of the 16S rRNA gene (1,300-1,500 bp). Sequencing results showed that the LAB isolates belonged to the genera *Lactobacillus* (L.), *Weissella* (W.), and *Pediococcus* (P.), respectively (data not shown). These isolates were identified at species and subspecies levels as *L. paraplantarum* (1 strain), *L. pentosus* (3 strains), *L. plantarum* (8 strains), *P. pentosaceus* (1 strain), *P. stilesii* (1 strain), and *W. halotolerans* (1 strain). Among 15 strains, 5 strains showed effective TMA reduction activity and these strains were tentatively named as *L. plantarum* SKD1 and SKD4, *L. paraplantarum* SKD15, *P. pentosaceus* SKD14, and *P. stilesii* SKD 11, based on the 16S rRNA gene sequence analysis.

These five LAB isolates were also screened for their performance regarding growth characteristics in carbon source using an API 50CH and 50CHL medium system. The results of biochemical identification showed similar results with 16S rRNA gene identification results (Table 1). All LAB isolates ferment different carbon sources including D-ribose, D-glucose, D-fructose, D-mannose, N-acetyl-glucosamine, amygdalin, arbutin, esculin, and salicin. Interestingly, the strain SKD 14 utilizes both D-xylose and D-tagatose, and the SKD 11 strain ferments an additional carbon source, D-lyxose. Other strains, SKD 1, 4, and 15, can utilize hexoses such as mannitol and sorbitol (Table 2). Ashmaig *et al*. [19] screened LAB from traditional Sudanese fermented camel’s milk and they reported similar results [20]. Although there are variations in the utilization of carbohydrate sources in the API 50 CH and CHL system, these biochemical tests are in agreement with 16S rRNA-based identification towards the characterization of these LAB strains [21].

**Fig. 1:**
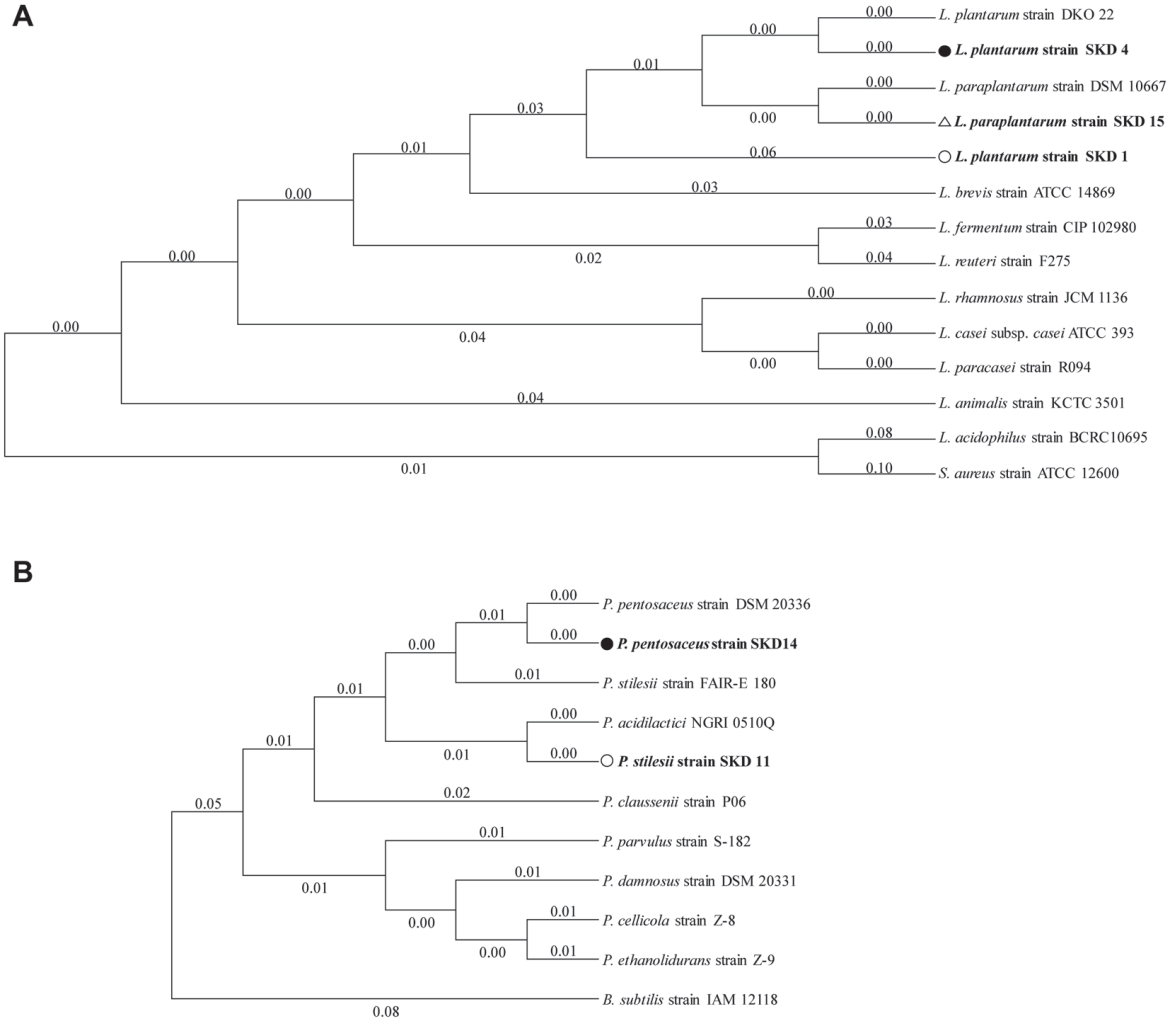
Neighbor-joining phylogenetic tree based on 16S rRNA gene sequences. The neighbor-joining phylogenetic tree shows the relationship between LAB isolates. (A) SKD 1, 4, and 15 (○, ●, and △) belong to *Lactobacillus* species. (B) SKD 11, and 14 (○, ●) belong to *Pediococcus* species. Numbers at behind strain names are accession numbers of the published sequences at NCBI.

In an effort to identify SKD 1, 4, 11, 14, and 15 isolates at the species level, molecular phylogenic analysis was conducted and the phylogenetic tree was constructed based on the 16S rRNA gene sequence from evolutionary distances by the neighbor-joining method (Figs. 1A and 1B). Based on the analysis result, (Fig. 1A), SKD 1, 4, and 15 strains belonged to the genus *Lactobacillus* sp. and included *L. plantarum*, *L. paraplantarum*, *L. brevis*, *L. fermentum*, *L. reuteri*, *L. rhamnosus*, *L. casei*, *L. paracasei*, *L. animalis*, and *L. acidophilus*. Similarly, the strains SKD 11 and 14 belonged to the genus *Pediococcus* sp. and included *P. pentosaceus*, *P. stilesii*, *P. acidilactici*, *P. claussenii*, *P. parvulus*, *P. damnosus*, *P. celicola*, and *P. ethanolidurans* (Fig. 1B). Thus, we concluded that the strains SKD 1, 4, and 15 clustered with a 16S rRNA gene sequence of *Lactobacillus* species (Fig. 1A), and SKD 11 and 14 clustered with a sequence of *Pediococcus* species (Fig. 1B). The GenBank accession numbers for the 16S rRNA gene sequences of isolates were given in Table 1.

### LAB Exhibiting TMA Reduction Efficacy

All five LAB strains, SKD 1, 4, 11, 14, and 15, showed significant reduction of TMA within 5 h of incubation and the values were 88.2%, 88.1%, 89.86%, 92.14%, and 91.33%, respectively. Furthermore, after 24 h of incubation, the reduction of TMA by strains SKD 11, 14, and 15 was found to be similar as reported during 5 h of incubation. However, the reduction of TMA by strains SKD 1 and 4 was found to be slightly decreased during 24 h of incubation (Table 1). Based on the above results, we concluded that the maximum reduction of TMA occurred by each LAB isolate within 5 h of incubation; however, there was no significant reduction during prolonged incubation. This might suggest that the enzyme present in the cell-free supernatant gets fully saturated with the available substrate and showed maximum catalytic activity within 5 h of incubation.

Boraphech and Triravetyan [13] screened several plant materials having TMA reduction activity and they reported *Sansevieria* sp. was the most effective among them. Similarly, it was reported that *Sanseveria kirkii* was the most effective species among Sanseveria sp., and the TMA reduction mechanism was found due to the adsorption to the wax stream [12]. These results indicate that microbial species and biological materials could be used as TMA reduction agents and can also be further applied to the seafood industry for deodorization [13]. This new approach to deodorization has advantages of being very fast, low cost, and eco-friendly. Therefore, we sought to verify the applicability of this biological TMA reduction approach to the fisheries industry by using spoiled fish samples.

### In Situ Reduction of TMA Content from Spoiled Fish by LAB Strains 

As shown in Table 3, TMA contents in spoiled fish were decreased by the treatment of five LAB strains (SKD1, 4, 11, 14, and 15). The five LAB strains showed TMA reduction activity and have stable performance and activity as compared to other LAB strains. The TMA reduction efficiency of the five LAB cell-free cultures’ supernatant was reported as 45%, 62%, 60%, 59%, and 52% by SKD 1, 4, 11, 14, and 15, respectively. The in situ results indicate that sample treated with LAB cell-free supernatant showed significant TMA reduction in the range of 45-62%, which is lower as compared to the LAB-treated TMA reduction (in the range of 88.17-92.87%.). The lesser amount of TMA reduction from fish treated with LAB cell-free supernatant might be due to the interaction of TMA with the secondary metabolites produced by LAB [22], which might counter the action of TMA degrading enzymes present in the supernatant. However, a future study is required to explain this mechanism. Although in situ reduction of TMA is lower as compared to the LAB-treated TMA reduction, a significant in situ TMA reduction suggests that these LAB isolates can be applied in the seafood industry to effectively reduce the off-odor of spoiled fish by decreasing TMA content.

Researchers in several studies have tried to remove TMA using various methods. Among them, it was reported that cellulose-based active carbon fiber, charcoal particles, and ortho-phosphoric acid were able to remove gases such as TMA and ammonia [7, 10, 21]. It was also reported that a continuous flow extraction using microbubbles combined with supercritical CO_2_ removed volatile compounds released from the fish sauce [8]. However, these previous methods have a limitation in that they are applicable only for liquid and not solid samples. In contrary to the previous methods, the present results obtained in this study strongly suggest LAB isolates and their cell-free supernatant are applicable to remove volatile compounds such as TMA from both liquid and solid samples. To the best of our knowledge, this is the first report on TMA reduction by metabolic activity of LAB isolates and its in situ application using spoiled fish samples.

In conclusion, the present study reported a new approach for the deodorization of TMA or fish off-odor by microbial treatment especially using LABs. In addition, LAB treatment was effective in the deodorization of spoiled fish samples. LAB strains that were isolated from Korean traditional fermented foods have been characterized by biochemical and 16S rRNA gene sequencing approaches. The identified LAB isolates were characterized as *L. plantarum* (SKD1 and 4), *P. stilesii* (SKD 11), *P. pentosaceus* (SKD14) and *L. paraplantarum* (SKD 15). Overall, the TMA reduction rate by these LAB isolates was ranged from 88.17 to 92.14% in 5 h reaction. Similarly, in 24 h reaction, the TMA reduction rate by LAB cell-free culture supernatant was ranged from 84.56%to 92.87%. Furthermore, the in situ application results also showed that LAB cell-free culture supernatant treatment significantly reduced TMA. However, a future study is required to test the TMA reduction ability of the LAB using different species of fish samples. Further study is also required to check the taste and flavor of the fish samples treated with LAB cell-free culture supernatant samples. Therefore, we suggested that the application of LAB or LAB cell-free supernatant will be economically and conveniently useful to reduce or retard TMA in the seafood handling process. The present study can be applied to all fishery products for the deodorization of fish off-odor using LAB due to the low cost and deodorization efficiency (in the range of 45-62%).

## Figures and Tables

**Table 1:** Identification of lactic acid bacteria (LAB) isolated from Korean traditional fermented foods and their TMA reduction efficacy.

Source	Strain No.	Identification by 16S rRNA gene sequences			TMA reduction efficacy of isolated LAB			

					Reduction efficiency (mg/l)		Reduction percentage (%)^1^	

		Identified strains	Similarity^2^	Gene Bank Accession No.	5 h reaction	24 h reaction	5 h reaction	24 h reaction
Fermented soybean block (meju)	SKD1	*Lactobacillus plantarum* strain CIP 103151	99%	MK785052	190.69 ± 2.94a*	224.53 ± 2.69a	88.24	84.72
	SKD4	*Lactobacillus plantarum* DSM 20314	99%	MK758000	191.87 ± 6.20a	226.77 ± 3.16a	88.17	84.56
Kimchi	SKD11	*Pediococcus stilesii* strain	99%	MK758076	151.33 ± 3.63b	152.06 ± 3.00b	90.67	89.65
Fermented kelp	SKD14	*Pediococcus pentosaceus* DSM 20336	99%	MK758078	127.46 ± 2.88c	104.70 ± 27.54d	92.14	92.87
	SKD15	*Lactobacillus paraplantarum* DSM 10667	100%	MK758083	118.43 ± 2.83d	134.70 ± 1.35c	91.70	90.83

^1^The percentage of TMA reduction was calculated based on the standard of TMA (final concentration of 0.3% v/v) prepared in MRS medium. The values obtained from the standard at 5 h and 24 h incubation were found to be 1,621.86 ± 23.43 and 1,469.04 ± 23.73 mg/l, respectively.

^2^Relationship based on the similarity of 16S rRNA gene sequences between isolates and reference strain.

^*^Values are presented as mean ± SD with three replicates. Data with different letters indicate significant difference at *p* < 0.05.

**Table 2:** Carbohydrates fermentation by lactic acid bacteria (LAB) isolated from traditional Korean fermented foods using API 50 CHL system.

Carbon sources	LAB isolates					Carbon sources	LAB isolates				
	
	SKD 1	SKD 4	SKD 11	SKD 14	SKD 15		SKD 1	SKD 4	SKD 11	SKD 14	SKD 15
Glycerol	-	-	-	-	-						
Erythritol	-	-	-	-	-	Salicin	+	+	+	+	+
D-arabinose	-	-	-	-	-	Cellobiose	+	+	-	+	+
L-arabinose	+	+	+	+	-	Maltose	-	+	+	+	+
D-ribose	+	+	+	+	+	Lactose	+	+	+	-	-
D-xylose	-	-	-	+	-	Melibiose	+	+	+	-	-
L-xylose	-	-	-	-	-	Sucrose	+	+	-	-	+
D-adonitol	-	-	-	-	-	Trehalose	-	-	-	+	+
Methyl-BD Xylopyranosicle	-	-	-	-	-	Inulin	+	+	-	-	-
D-Galactose	+	+	+	+	-	Melezitose	+	-	+	-	+
D-Glucose	+	+	+	+	+	Raffinose	-	-	-	-	-
D-fructose	+	+	+	+	+	Starch	-	-	-	-	-
D-mannose	+	+	+	+	+	Glycogen	-	-	-	-	-
L-sorbose	-	-	-	-	-	Xylitol	+	+	+	-	-
Rhamnose	+	+	-	-	-	Gentiobiose	+	+	-	+	+
D-ulcitol	-	-	-	-	-	D-Turanose	-	-	-	-	-
Inositol	-	-	-	-	-	D-Lyxose	-	-	+	-	-
Mannitol	+	+	-	-	+	D-Tagatose	-	-	-	+	-
Sorbitol	+	+	-	-	+	D-Fucose	-	-	-	-	-
α-methyl-D-mannoside	-	-	-	-	-	L-Fucose	-	-	-	-	-
α-methyl-D-glucoside	-	+	+	-	+	D-Arabitol	-	-	-	-	-
N-acetyl-glucosamine	+	+	+	+	+	L-Arabitol	+	+	-	-	-
Amygdalin	+	+	+	+	+	Gluconate	-	-	-	-	+
Arbutin	+	+	+	+	+	2-keto-gluconate	-	-	-	-	-
Esculin	+	+	+	+	+	5-keto-gluconate	-	-		-	-

Readings were done under anaerobic conditions after 24 h at 37°C. +, Positive reaction, -, Negative reaction.

**Table 3:** Reduction of trimethylamine (TMA) contents in the spoiled fish samples (*Trichiurus lepturus*) byLAB cell-free treatment.

Sample	TMA (mg/l)	Reduction efficiency (%)
Control (no treatment) Treated with SKD1	384 ± 33^a*^ 212 ± 6b	100 45
Treated with SKD4	144 ± 6^d^	62
Treated with SKD11	152 ± 16^d^	60
Treated with SKD14	157 ± 3^d^	59
Treated with SKD15	185 ± 9^c^	52

1 g of fish was homogenized, and incubated at 25°C for 48 h and then treated with or without 4 ml of cell-free supernatant.

^*^Values are presented as mean ± SD with three replicates. Data with different letters indicate significant difference at *p* < 0.05.
